# Supplementary material for: Repression of CADM1 transcription by HPV type 18 is mediated by three-dimensional rearrangement of promoter-enhancer interactions
Source: PLoS Pathog. 2025 Jan 27;21(1):e1012506. doi: 10.1371/journal.ppat.1012506 (PMC11801731; doi:10.1371/journal.ppat.1012506)
Supplement: S1 Table — Table shows total number of reads per sample alongside number of uniquely mapped reads, reads mapped to the HPV18 genome (AY262282.1) and the number of HPV-host fusion transcripts identified. (DOCX) [file ppat.1012506.s001.docx]

**S1 Table:**

| Sample | Total fragments (millions) | Uniquely mapped reads in millions (% of total) | HPV uniquely mapped fragments (% of total) | HPV-human fusion transcripts (% of total HPV) |
| --- | --- | --- | --- | --- |
| HFK-1 | 41.8 | 37 (88.5) | 2 (0.000005) | - |
| HPV18-1 | 45.5 | 39.6 (87.1) | 28971 (0.064) | 7 (0.024) |
| HFK-2 | 46.5 | 39.7 (85.3) | - | - |
| HPV18-2 | 50.8 | 43.8 (86.2) | 21712 (0.043) | 68 (0.31) |
| HFK-3 | 36.4 | 31.2 (85.8) | - | - |
| HPV18-3 | 37.1 | 31.4 (84.6) | 25851 (0.070) | 55 (0.21) |
| HFK-4 | 43.1 | 37.3 (86.5) | - | - |
| HPV18-4 | 37.7 | 32.7 (86.9) | 28638 (0.076) | 30 (0.11) |
| HFK-5 | 51.6 | 43.7 (84.8) | 1 (0.000002) | - |
| HPV18-5 | 41.7 | 35.5 (85) | 18403 (0.044) | 9 (0.049) |
| HFK-6 | 43.5 | 37.1 (85.4) | - | - |
| HPV18-6 | 35.7 | 27 (75.7) | 16880 (0.0473) | 16 (0.095) |
