# Supplementary figures and images for: Repression of CADM1 transcription by HPV type 18 is mediated by three-dimensional rearrangement of promoter-enhancer interactions
Source: PLoS Pathog. 2025 Jan 27;21(1):e1012506. doi: 10.1371/journal.ppat.1012506 (PMC11801731; doi:10.1371/journal.ppat.1012506)

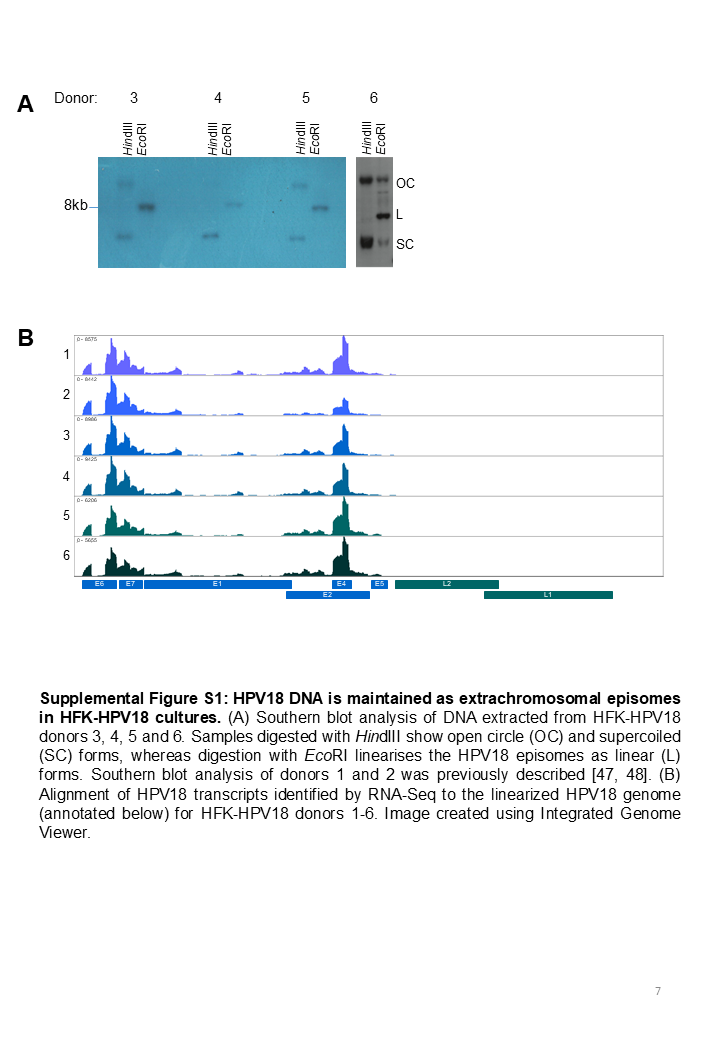

Supplement: S1 Fig — (A) Southern blot analysis of DNA extracted from HFK-HPV18 donors 3, 4, 5 and 6. Samples digested with HindIII show open circle (OC) and supercoiled (SC) forms, whereas digestion with EcoRI linearises the HPV18 episomes as linear (L) forms. Southern blot analysis of donors 1 and 2 was previously described [48,49]. (B) Alignment of HPV18 transcripts identified by RNA-Seq to the linearized HPV18 genome (annotated below) for HFK-HPV18 donors 1-6. Image created using Integrated Genome Viewer. (TIF) [file ppat.1012506.s007.tif]

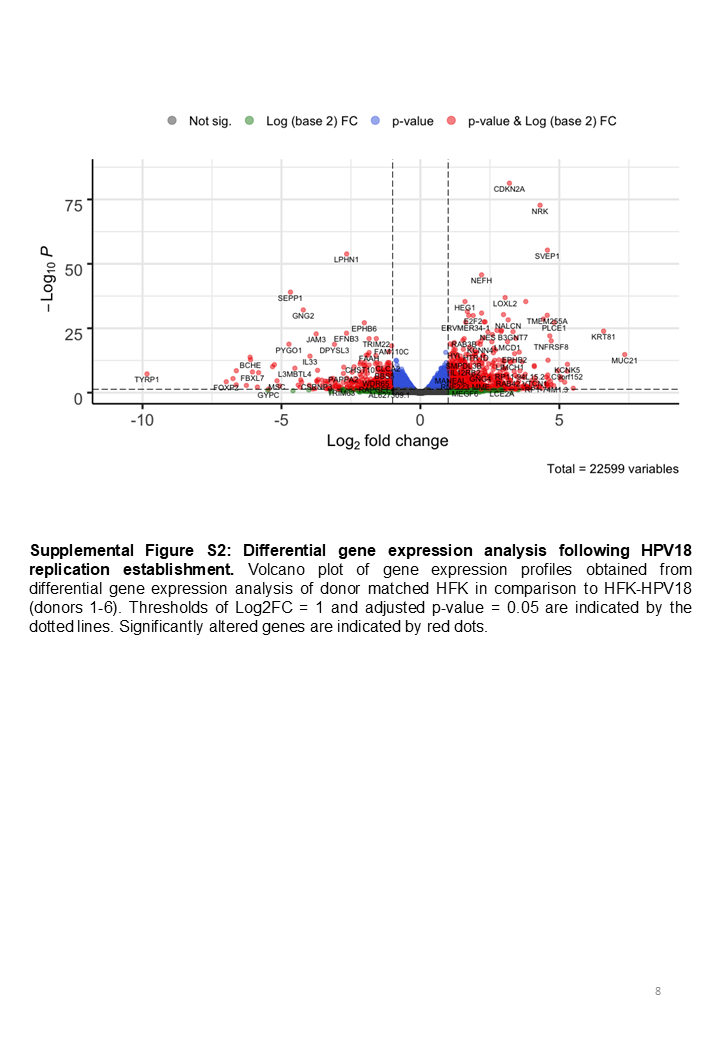

Supplement: S2 Fig — Volcano plot of gene expression profiles obtained from differential gene expression analysis of donor matched HFK in comparison to HFK-HPV18 (donors 1-6). Thresholds of Log2FC = 1 and adjusted p-value = 0.05 are indicated by the dotted lines. Significantly altered genes are indicated by red dots. (TIF) [file ppat.1012506.s008.tif]

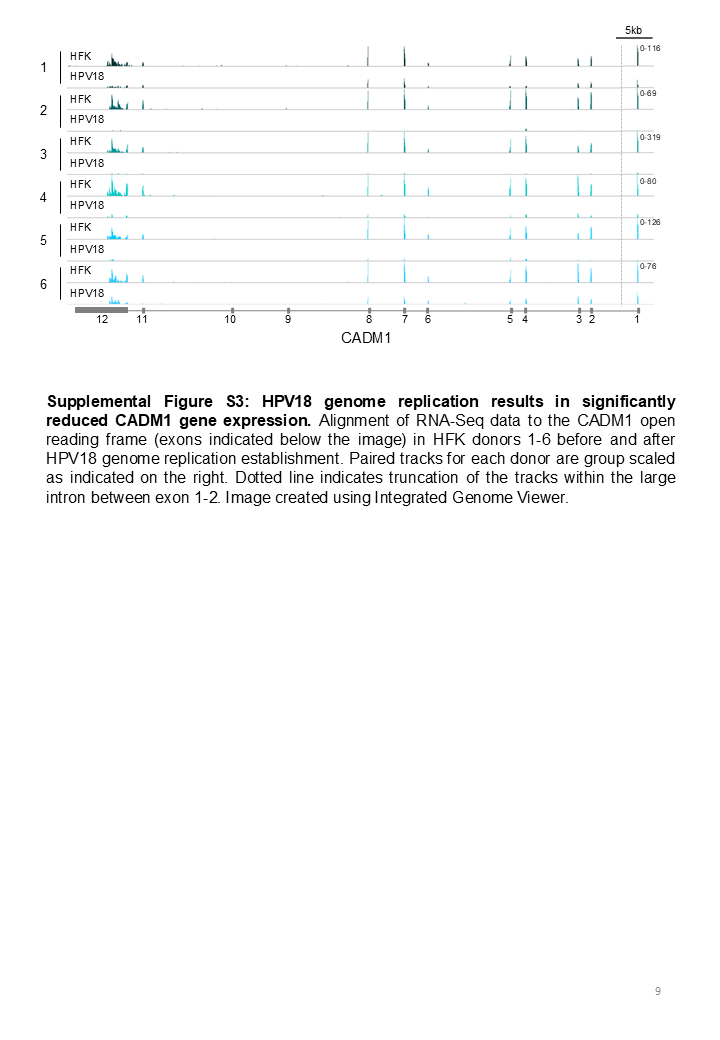

Supplement: S3 Fig — Alignment of RNA-Seq data to the CADM1 open reading frame (exons indicated below the image) in HFK donors 1-6 before and after HPV18 genome replication establishment. Paired tracks for each donor are group scaled as indicated on the right. Dotted line indicates truncation of the tracks within the large intron between exon 1-2. Image created using Integrated Genome Viewer. (TIF) [file ppat.1012506.s009.tif]

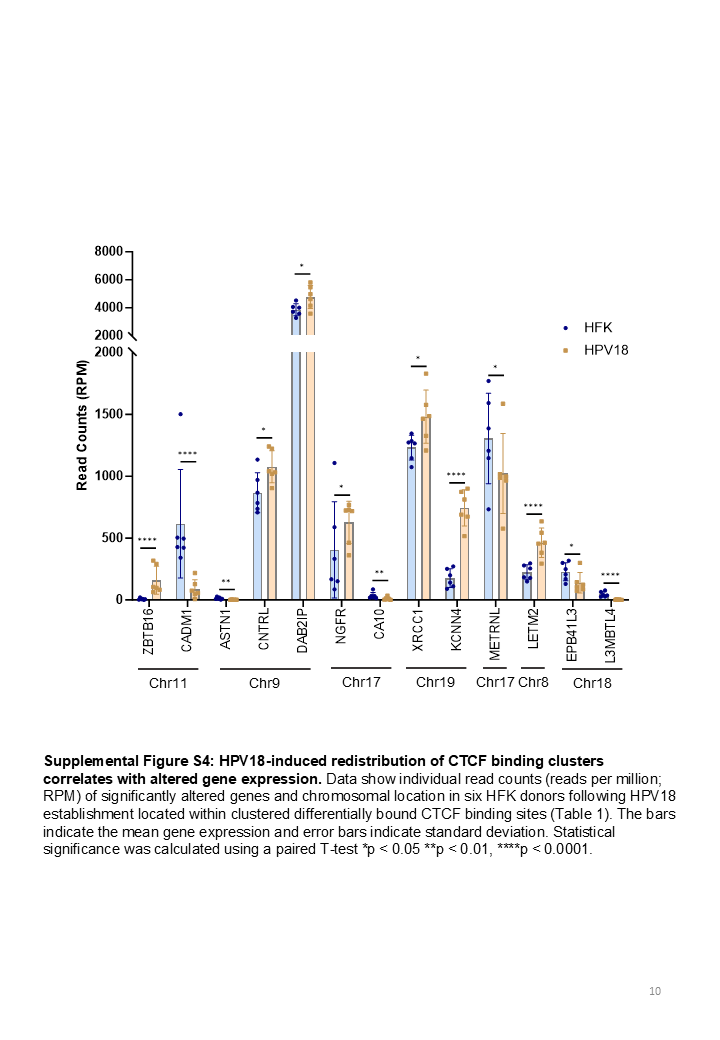

Supplement: S4 Fig — Data show individual read counts (reads per million; RPM) of significantly altered genes and chromosomal location in six HFK donors following HPV18 establishment located within clustered differentially bound CTCF binding sites (Table 1). The bars indicate the mean gene expression and error bars indicate standard deviation. Statistical significance was calculated using a paired T-test *p < 0.05 **p < 0.01, ****p < 0.0001. (TIF) [file ppat.1012506.s010.tif]
