# Supplementary material for: Repression of CADM1 transcription by HPV type 18 is mediated by three-dimensional rearrangement of promoter-enhancer interactions
Source: PLoS Pathog. 2025 Jan 27;21(1):e1012506. doi: 10.1371/journal.ppat.1012506 (PMC11801731; doi:10.1371/journal.ppat.1012506)
Supplement: Attachment — Submitted filename: Response to reviewers.docx [file ppat.1012506.s014.docx]

**Reviewer #1**

1). What happens to CAMD1 or ZBTB16 expression if CTCF is knocked down? If CTCF is an essential gene, then short-term knockdowns with siRNAs may work. ZBTB16 can be assayed by RT-qPCR since the antibody is not very good.

We have previously considered that knockdown of CTCF could be used to demonstrate the dependency of CADM1/ZBTB16 expression on the arrangement of CTCF binding within this locus. However, previous studies have demonstrated that acute depletion of CTCF, either by siRNA-mediated mRNA depletion or auxin-induced protein degradation, has highly variable effects on transcription and occupancy of individual CTCF binding sites (e.g. PMID: 36852270, 36471071, 34429148). Even at auxin-induced degradation of CTCF to levels of <1% of the total, almost 30% of CTCF binding sites detected by ChIP-Seq remain occupied. The persistence of CTCF binding and intensity of the peaks detected did not correlate in this study, indicating that other factors are at play in dictating stability of CTCF occupancy. However, a significant correlation was found with persistent CTCF occupancy and long-range chromatin interactions including promoter-enhancer interactions. We therefore ruled out using acute depletion of CTCF to validate our observation that CTCF binding loss correlate with CADM1/ZBTB16 gene expression changes as the results are likely to be impossible to interpret.

2). Is there any looping between the HPV 18 CTCF site and CADM1?

Analysis of our 4C data by mapping to the HPV18 genome revealed that no sequencing reads aligned to the HPV18 genome in either donor tested. These data indicate that the HPV18 genome, maintained predominantly in an episomal state in our HFK donor cells, does not physically interact with the viewpoint centred on the CADM1 transcriptional start site used in our 4C experiment.

This finding has been added to our manuscript (lines 357-359).

3). While there was no difference in the total CTCF-bound peaks, were there novel CTCF binding sites within the HPV 18 + donors? Are the differentially bound CTCF peaks representative of novel sites or just altered binding (ie. Where the CTCF reads were at or below the input control level in one sample but significantly above that threshold in another)?

To answer this query, we have now used REMAP as a collection of previously described CTCF binding sites from experimental data (<https://remap.univ-amu.fr/>). The annotation of all non-redundant experimentally validated CTCF binding sites in the human genome (<https://remap.univ-amu.fr/target_page/CTCF:9606>) was then cross compared to the peaks identified in our experimental samples. These data have been added as a new column in the Total CTCF peaks tab in Table S4 named ‘Remap’. If the annotation is “yes”, the peak has been previously described if “no”, it is a newly identified peak. Using this method, 99.0% of the peaks have been previously described.

Analysis of the 902 differential peaks (HPV18 vs UT) in Table S4 using the same method revealed that 97.9 % of the detected peaks have been previously described. Only 2.1% are not overlapping with database peaks, none of which are located at the CADM1 locus on Chr 11.

These new data indicate that most of the differentially bound peaks in HPV18-HFK compared to HFK are previously annotated bone fide CTCF binding sites, rather than novel sites, that are differentially occupied in response to HPV18 replication establishment.

We have also added information on whether a differential peak is only detected in the HPV18 genome containing cells and not in the control HFK samples. These new data are in the column named “only_in_HPV18” in Table S4 (Differential Peaks tab). Using this analysis, only 16.9% of differential peaks are found in the HPV18 samples only.

These findings have been added to the text of our manuscript (lines 207 to 216) and new data added in Table S4.

4). Is 100kb the standard/accepted genomic size to associate CTCF binding sites with gene expression? Since about 45% of your CTCF binding sites in both HPV 18 + and - cells were in gene bodies, proximal promoter, or distal promoter regions, why not use these binding sites (and any differential CTCF binding between HPV + vs -) to integrate the differential gene expression data?

Thank you for the suggestion. We have now calculated the enrichment of differentially expressed genes directly overlapping differential CTCF binding sites and added the following sentence to the corresponding section (lines 252-255):

“When focusing on genes that directly overlapped with differential CTCF binding sites (distance = 0 in Table S5), we observed a 2.7-fold enrichment, with 38 differentially expressed genes (3.9%) compared to 323 (1.43%) of all genes, yielding a Hypergeometric test p-value of 7.3 × 10^⁻9^.”

5). In Table 1, are the differentially expressed genes representative of the average of the six donor samples (+ and -), or is the differential expression true for each donor? The consistent differential expression of ZBTB16 and CADM1 in each donor is striking. Clarification of whether some of the differentially expressed genes listed in Table 1 follow the same trend would support the mechanistic impact of differential CTCF binding.

We agree that the addition of these data would enhance our manuscript. We have therefore added a new Figure S4 showing the variation in read counts per million of the significantly altered genes shown in Table 1 in the six donor HFKs tested.

6). Is the % CpG methylation of CADM1 TSS in the HPV 18 genome containing cells different than that seen in the HPV negative donors? This would seem to be a more direct comparison to determine hyper- versus hypomethylation in different cell lines.

We agree that it would be better to compare the % CpG methylation at the CADM1 locus in HFK donors before and after HPV18 transfection. We have therefore sequenced DNA extracted from the three HFK donor cell cultures using the adaptive Nanopore sequencing method described in our manuscript. These new data are included in Figure 6C. No significant difference was observed in %CpG methylation at the CADM1 TSS between the three HFK donors analysed and the matched HPV18-HFKs cultures (p = 0.078).

Discussion of these findings have been added to the manuscript (lines 391 – 393).

Reviewer #2: is it possible to test whether the changes in CADM1 and ZBTB16 expression in HPV18-expressing cells can be reversed by manipulating CTCF expression (RNAi, CRISPRi)

See response to Reviewer 1, point 1 above.

Reviewer #3: The introduction cites repeatedly that the prior literature that HPV infection and cancer leads to increased levels of CTCF protein reported to be post-transcriptional. It would be useful if a specific comment about the levels of CTCF in the transcriptome of the normal and infected cells in the current experiments could be noted. In addition, it would be helpful to perform western blots of CTCF to evaluate if levels were increased in the infected cells. It is not clear if the current experiments were similar to the earlier reports from this lab (reference #30).

We have now added the requested data providing analysis of CTCF transcript and protein abundance before and after HPV18 replication. The data confirm no change in CTCF transcript abundance in the HFK donors used in this study. However, Western blot analysis of the five out of six donors, that we had material available for, revealed an inconsistency in the previously reported increase in CTCF protein abundance induced by HPV18 replication. On average, there was a small but insignificant increase in CTCF protein abundance, but this was highly variable between the HFK donors analysed. These new data have now been included in the revised manuscript as Fig 1D and described in lines 191-195.

**Minor Issues:**

**Reviewer #2:** 1.The font size in many of the figures should be increased to make sure that they remain legible.

The font size has been increased or set to bold in the revised figures where necessary

2.In Figure 3B "Read counts" should be replaced with "CADM1 Read Counts" and ditto for "Fold Expression"

This has been changed as requested

3.Reference 44 should receive a bit more discussion (lines 404 to 407)

This reference has been discussed in greater detail. (lines 434-444).

4.Something is missing in the sentence "GSEA highlighted ..." (lines 410-411)

This sentence has been modified (lines 448-450)

5.On line 416 delete "REF"

(REF) has been deleted

Reviewer #3: The redistribution of CTCF binding sites in the normal vs infected cells was reported in the text (lines 194-200) as a significant shift from genebody (40%) and promoter (14%) in normal cells toward intergenic and gene desert regions in infected cells. However, I find that the Venn diagrams in Figure 2C do not effectively illustrate this change as I am forced to visually estimate the size of the pie slice. Perhaps a bar graph or another type would be clearer.

We have now added bar graph below each pie chart (Fig, 2C) which depict the absolute number of peaks in each category.

The use of nanopore sequencing to identify DNA methylation was a very powerful approach to address the question of the impact of HPV18 infection on DNA methylation and if this contributed to changes in CTCF binding. However, it was not clear from the main text and methods if both the normal and infected cells were sequenced or was it just the infected cells from three donors. If just the infected cells were sequenced, how were the changes assessed on Figure 6? Furthermore, the discussion section that describes these effects (lines 454-463) is not clear. If the CADM1 region is hypermethylated in HPV-driven cancer, how does this relate to the regions that are described as hypomethylated in the manuscript?

The original manuscript included analysis of %CpG methylation at the CADM1 and upstream APO5 regions of just the HPV infected donors. We have now included the same analysis of the uninfected matched donors for comparison. These new data are included in Figure 6C. No significant difference was observed in %CpG methylation at the CADM1 TSS between the three HFK donors analysed and the matched HPV18-HFKs cultures (p = 0.078). These findings have been included to the manuscript (lines 392 – 394).

We have now edited the discussion to clarify that the high CpG methylation at the CADM1 TSS observed in HPV+ cancers occurs later in the progression of disease than our study is currently modelling (lines 500-518).
